# Morphological Changes of Paulownia Seedlings Infected Phytoplasmas Reveal the Genes Associated with Witches' Broom through AFLP and MSAP

**DOI:** 10.1371/journal.pone.0112533

**Published:** 2014-11-26

**Authors:** Xibing Cao, Guoqiang Fan, Zhenli Zhao, Minjie Deng, Yanpeng Dong

**Affiliations:** 1 Institute of Paulownia, Henan Agricultural University, Zhengzhou, Henan, P. R. China; 2 College of Forestry, Henan Agricultural University, Zhengzhou, Henan, P. R. China; Institute of Crop Sciences, China

## Abstract

Paulownia witches' broom (PaWB) caused by phytoplasma might result in devastating damage to the growth and wood production of *Paulownia*. To study the effect of phytoplasma on DNA sequence and to discover the genes related to PaWB occurrence, DNA polymorphisms and DNA methylation levels and patterns in PaWB seedlings, the ones treated with various concentration of methyl methane sulfonate (MMS) and healthy seedlings were investigated with amplified fragment length polymorphism (AFLP) and methylation-sensitive amplification polymorphism (MSAP). Our results indicated that PaWB seedlings recovered a normal morphology, similar to healthy seedlings, after treatment with more than 20 mg·L^−1^ MMS; Phytoplasma infection did not change the Paulownia genomic DNA sequence at AFLP level, but changed the global DNA methylation levels and patterns; Genes related to PaWB were discovered through MSAP and validated using quantitative real-time PCR (qRT-PCR). These results implied that changes of DNA methylation levels and patterns were closely related to the morphological changes of seedlings infected with phytoplasmas.

## Introduction

*Paulownia*, one of the fastest growing trees in the world, is native to China. In recent years, it has been introduced in many other countries [1]. Due to the biological and wood properties of this tree, it is ideal for use in house construction, pulp and paper, furniture, farm implements and handicrafts [2], even as an intercropping species in modern agroforestry and environmental protection [3], [4].

Paulownia witches' broom (PaWB), the most destructive infectious disease of *Paulownia*, is caused by phytoplasma of Aster Yellows group ‘*Candidatus* Phytoplasma asteri’ [5]. Paulownia trees with this disease show numerous morphological symptoms, including axillary bud germination, shorter internodes and smaller leaf etiolation, resulting in significant decline in vigor and growth of the tree, even premature death of the tree [3]. Since the 1970s, abundant of researches about the way and route of pathogen transmission [6], the prevention and control of insect vectors [7], [8], and physiological and biochemical variation of Paulownia during the occurrence of PaWB were carried out, and several metabolic pathways related to PaWB were reported by Liu et al. [9] and Mou et al. [10]. Although these researches are helpful to understand the interaction of Paulownia and phytoplasma, the molecular mechanism of PaWB occurrence is still remain elusive.

DNA methylation is a widespread epigenetic modification, mainly occurs at gene promoter, or transcribed regions [11]–[13], playing an important role in regulation of gene expression mediating variation of plant morphology [14]. For example, flower abnormalities caused by the tomato stolbur phytoplasma were correlated with gene-specific demethylation [15]. In Paulownia, previous studies have shown that the global DNA methylation level of PaWB seedlings (PS) was lower than that of healthy seedlings (HS) [16]. MMS, a DNA methylating agent, can modify guanine to 7-methylguanine and adenine to 3-methyladenine, and increase the methylation level of 5-cytosine [17]. Our previous results showed that the PS could recover a healthy morphology by treatment with suitable concentration of MMS, in which the phytoplasma could be removed [8], [18], [19]. However, the relationship between morphological changes of PaWB seedlings and DNA methylation has not been studied.

Amplified fragment length polymorphism (AFLP) and methylation-sensitive amplification polymorphism (MSAP) are efficient and reliable methods to detect DNA polymorphism and DNA methylation, respectively [20], [21]. Here, with these two approaches, the variations of DNA polymorphisms and DNA methylation in PS, the ones treated with MMS and HS were investigated in order to reveal the genes associated with PaWB. The results will provide new insights for further studies into the mechanism of PaWB.

## Materials and Methods

### Plant materials

Healthy *Paulownia fortunei* and PaWB tissue cultured seedlings were obtained from the Institute of Paulownia, Henan Agricultural University, Zhengzhou, China. The two types of tissue cultured seedlings were first cultivated on 1/2 Murashige - Skoog (MS) medium [22] for 30 days, before uniform terminal buds of about 1.5 cm in length from the PS were transferred into 100 mL flasks containing 1/2 MS medium (40 mL) including 25 mg·L^−1^ sucrose and 8 mg·L^−1^ agar (Sangon, Shanghai, China) with 0 mg·L^−1^MMS, 20 mg·L^−1^MMS (PS-20), 60 mg·L^−1^MMS (PS-60), 100 mg·L^−1^MMS (PS-100). The terminal buds of the HS were transferred into 1/2 MS medium without MMS. For each treatment, 60 terminal buds were planted into 20 flasks, each treatment was performed in triplicate. All samples were cultured initially at 20°C in the dark for 5 days. Thereafter, they were transplanted at 25±2°C and a light intensity of 130 µmol·m^−2^·s^−1^ with a 14∶10 h (light/dark) photoperiod. The method of morphological observation was performed according to Fan et al. [8]. Thirty days after the beginning of transplantation, terminal buds of 1.5 cm in length, growing in consistent condition, were sheared from the different seedlings, then immediately frozen in liquid nitrogen and stored at −80°C.

### Nucleic acid extraction

Total DNAs were extracted from the terminal buds of different samples according to the cetyl trimethylammonium bromide (Beijing Chemical Co., Beijing, China) method, as described by Zhang et al. [23], RNase (Invitrogen, Carlsbad, CA, USA) was used to render the DNA free of genomic RNA contamination. Total RNAs were extracted following by an Aidlab total RNA extraction kit (Aidlab, Beijing, China). RNase free - DNase I (Invitrogen) was used to render the RNA free of genomic DNA contamination. The DNAs and RNAs were assessed with NanoDrop 2000 (Thermo Scientific, Wilmington, DE, USA).

### PaWB phytoplasma detection

PaWB phytoplasma was detected by nested-PCR as described by Lee et al. [24]. The PCR procedure and the method of agarose gel electrophoresis were performed by Fan et al. [8].

### AFLP and MSAP analysis

The AFLP digestion reaction comprised 3 U *Pst*I and 3 U *Mse*I (Li-COR, Co., Lincoln, NE, USA), the pre-amplification and selective amplification reaction conditions and the method of electrophoresis were adapted from Cao et al. [25]. The AFLP adapter and selective amplification primer sequences are listed in Table S1.

The MSAP experiment comprised two digestion reactions, the first digestion reaction included 16 U of *Eco*RI (TaKaRa, Dalian, China) plus 10 U of *Msp*I (TaKaRa), the second digestion reaction was the same as the first digestion except the *Hpa*II (TaKaRa) instead of *Msp*I. The MSAP pre-amplification and selective amplification reaction conditions and the method of electrophoresis were followed by Cao et al. [26]. The MSAP adapter and selective amplification primer sequences are listed in Table S2.

### Data analysis

After silver staining, only clear and reproducible bands were scored, where the presence of a band was scored as “1” and the absence was scored as “0”. For the MSAP analysis, the bands were scored according to the presence or absence of the bands in the products of *Eco*RI/*Hpa*II (H) and *Eco*RI/*Msp*I (M) digestions in different samples, according to these bands on the electrophoresis gels, the DNA methylation could be divided into three classes: class I indicated no methylation (the bands were present in both H and M), class II presented DNA hemi-methylation (the bands were present in H but absent in M), class III showed the DNA fully methylation (the bands were absent in H but present in M). Compared with the bands of PS, the DNA methylation patterns of PS treated with MMS or HS were classified into DNA methylation polymorphism and monomorphism. The DNA methylation polymorphisms included type A (DNA methylation), type B (DNA demethylation) and type C (uncertain DNA methylation). Among them, A_1_ and A_2_ were regarded as DNA de novo methylation (the bands were present in both H and M in PS, but only in H or M in the ones treated with MMS or HS), A_3_ and A_4_ were regarded as DNA hypermethylation (the bands were present only in H or M in PS, but absent in both H and M in the ones treated with MMS or HS). Type B (B_l_, B_2_, B_3_ and B_4_) showed DNA demethylation, the bands were the opposite to type A. Type C represented uncertain DNA methylation (the DNA methylation bands could not be determined between PS and the ones treated with MMS or HS). Type D (D_1_, D_2_ and D_3_) represented monomorphism (the status of the bands in M and H were the same in PS and the ones treated with MMS or HS). The statistical formulates used to score the bands were as follows: total DNA methylation level (%)  =  [(class II+class III)/(class I+class II+class III)]×100; DNA methylation polymorphism (%)  =  [(A+B+C)/(A+B+C+D)]×100; DNA methylation monomorphism (%)  =  [D/(A+B+C+D)]×100.

### DNA methylation patterns in different seedlings

**Figure 1:**
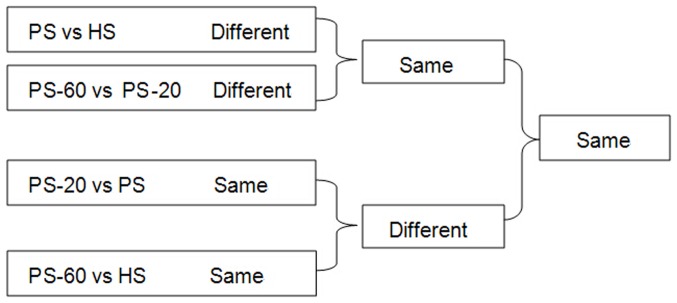
Comparison schemes of the different seedlings.

In order to identify the DNA methylation patterns related to PaWB, we compared the DNA methylation patterns in different seedlings (the seedlings in different morphology and the seedlings in the same morphology) (Figure 1). First, we compared the DNA methylation patterns in different morphological seedlings, in the PS vs. HS comparison, the factors for different DNA methylation patterns referred to PaWB and plant development difference (PDD); In the PS-60 vs. PS-20 comparison, the factors referred to PaWB, PDD and MMS treatments difference (MMST). In order to depart these factors from PaWB, we further picked out the same DNA methylation patterns in these two comparisons results. Obviously, the DNA methylation patterns involved in MMST were ruled out, and the DNA methylation patterns involved in PaWB and PDD were reserved. Second, we compared the DNA methylation patterns in same morphological seedlings, in the PS vs. PS-20 comparisons, the factors for same DNA methylation patterns involved in PaWB and PDD, in the PS-60 vs. HS comparisons, the factor only involved in PDD. In order to rule out the interference of PDD from the DNA methylation related to PaWB, we further reserved the different DNA methylation pattern in these two comparisons results, it was clear that the DNA methylation patterns involved in PDD were ruled out, and PaWB was reserved. At last, the same DNA methylations patterns related to PaWB were obtained from the first and second comparisons.

### Isolation and sequencing of polymorphic methylated fragments

Clear and reproducible bands from DNA methylation patterns related to PaWB were cut carefully with a clean blade and recovered using a UNIQ-10 Column DNA Gel Extraction Kit (Sangon, Shanghai, China), following the manufacturer's instructions. The DNA fragments were reamplified with the same selective primer combinations. The purified DNA was ligated into the pMD18-T easy vector (TaKaRa) and sequenced. The sequences obtained were analyzed using the Blastx programs at the NCBI website (http://www.ncbi.nlm.nih.gov/).

### Quantitative Real Time-PCR (qRT-PCR) analysis

The expressions of candidate genes were determined using qRT-PCR. First-strand cDNA was synthesized using an iScript cDNA Synthesis Kit (Bio-Rad, Hercules, CA, USA), according to the manufacturer's instructions. The PCR reaction contained SsoFast Supermix 10 µL (Bio-Rad), forward primer 0.4 µM (Sangon), reverse primer 0.4 µM (Sangon) and cDNA 1 µL, in a total volume of 20 µL. The qRT-PCR reaction mixture was run on a CFX96TM Real-Time PCR Detection System (Bio-Rad), starting with 95.0°C, 1 min; Then 40 cycles of 95.0°C for 10 s and 55.0°C for 15 s. 18SrRNA served as the internal reference gene. The results were analyzed using the 2^−ΔΔCt^ method [27], each qRT-PCR analysis was performed in triplicate. The primers used for the quantification of gene expression are listed in Table S3. Statistical analysis was performed using SPASS 19.0 (SPASS, Inc., Chicago, IL, USA).

## Results

### Morphological changes of different seedlings of the *P. fortunei* plants

**Figure 2:**
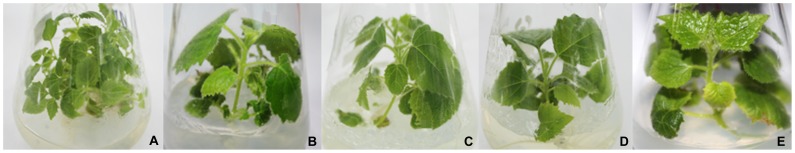
Changes of the morphology of PaWB seedlings with MMS treatment. A: PaWB seedlings (PS); B: PS-20; C: PS-60; D: PS-100; E: Healthy seedlings (HS).

Morphological changes of PS showed that the seedlings infected phytoplasma could recover a healthy morphology after MMS treatment (Figure 2). The small, light yellow leaves without seta turned into green leaves with seta, and the short internodes changed into normal internodes. Among these morphologic changes, only very tiny axillary buds were discovered in the PS-20, which disappeared in the PS-60 and PS-100 (Table 1). In addition, rooting rates increased significantly with the extension of incubation time at the same MMS concentration, but declined with MMS concentration increasing (*p*<0.05), simultaneously, the time of the first root of PS was also delayed with MMS concentration increasing. These results indicated that the PS could recover a healthy morphology after treatment with a suitable concentration of MMS.

**Table 1:** Development of MMS treated seedlings.

MMS concentrations	Rooting ratio/%			Rooting	Axillary	Leaves color and internodes	Terminal crown
/(mg·L^−1^)	10d	20d	30d	time/d	crowns		grow
0	90.0a	100a	100a	6	Yes	Small, light yellow leaf without seta and short internodes	Expand
20	75.0b	90a	100a	7	Yes	Green leaf with seta and normal internodes	Normal
60	30.0c	75.0b	86.7b	12	None	Green leaf with seta and normal internodes	Normal
100	0d	11.1c	31.7c	17	None	Green leaf with seta and normal internodes	Normal
HS	100a	100a	100a	5	None	Green leaf with seta and normal internodes	Normal

HS: Healthy seedlings. The different letters within a column indicate significant difference, while the same letters within a column indicate no significant difference (*p*<0.05).

### PaWB phytoplasma detection

**Figure 3:**
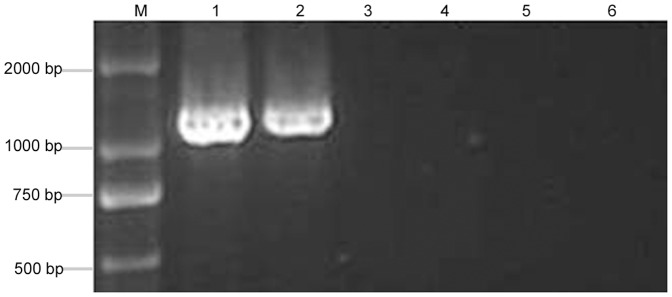
16S rDNA amplification in PaWB seedlings with MMS treatment. 1: PS; 2: PS-20; 3: PS-60; 4: PS-100; 5: HS; 6: ddH_2_O; M: DNA Marker.

To detect PaWB phytoplasma in PS and the ones treated with MMS, nested-PCR was performed to detect 16SrDNA of phytoplasma by universal phytoplasma primers. The result showed that the specific 1.2 kb fragments of phytoplasma were only detected in the PS and PS-20 (Figure 3), but the specific band was not detected in the healthy morphology seedlings, such as PS-60, PS-100, and HS. These results illustrated that the key reason for the recovery was because MMS removed the PaWB phytoplasma.

### Paulownia DNA sequence polymorphism

To study the effect of the phytoplasma infection on DNA sequence of Paulownia seedlings, Paulownia DNA polymorphisms of PS, the ones treated with MMS and HS were detected with 96 pairs of AFLP primer combinations (Figure S1). The results showed that no polymorphic DNA fragments were amplified by the same primer combinations, and the sizes of the fragments were identical for each seedling, showing that phytoplasma infection did not change the DNA sequence at AFLP level.

### DNA methylation variations

#### Variations of DNA methylation levels

DNA methylation level of PS significantly increased with MMS concentration increasing (*p*<0.05) (Table 2). The DNA methylation levels of PS, PS-20, PS-60 and PS-100 were 26.01%, 29.33%, 32.29% and 33.59%, respectively, and the DNA methylation level of HS was 35.97%. This finding indicated that the DNA methylation levels of PS treated with MMS were higher than that of PS, but lower than that of HP. This implied that, to some extent, variations of DNA methylation levels of the PS and the ones treated with MMS were associated with the morphological changes.

**Table 2:** Changes of DNA methylation levels in PaWB seedlings with MMS treatment.

MMS concentration/(mg·L^−1^)	Total amplified bands^a^	Band of class I	Band of class II	Band of class III	Total methylated bands^b^	Methylation level/%^c^
0	2691	1991	247	453	700	26.01a
20	2713	1919	257	539	796	29.33b
60	2589	1753	283	553	836	32.29c
100	2477	1645	295	537	832	33.59d
HS	2357	1509	283	565	848	35.97e

a : Total amplified bands  =  band of class I+ band of class II+ band of class III;

b : total methylated bands  =  band of class II+ band of class III;

c : methylation level (%)  =  (total methylated bands)/(total amplified bands)×100; HS: Healthy seedlings. The different letters within a column indicate significant difference, while the same letters within a column indicate no significant difference (*p*<0.05).

#### Variations of DNA methylation patterns

**Figure 4:**
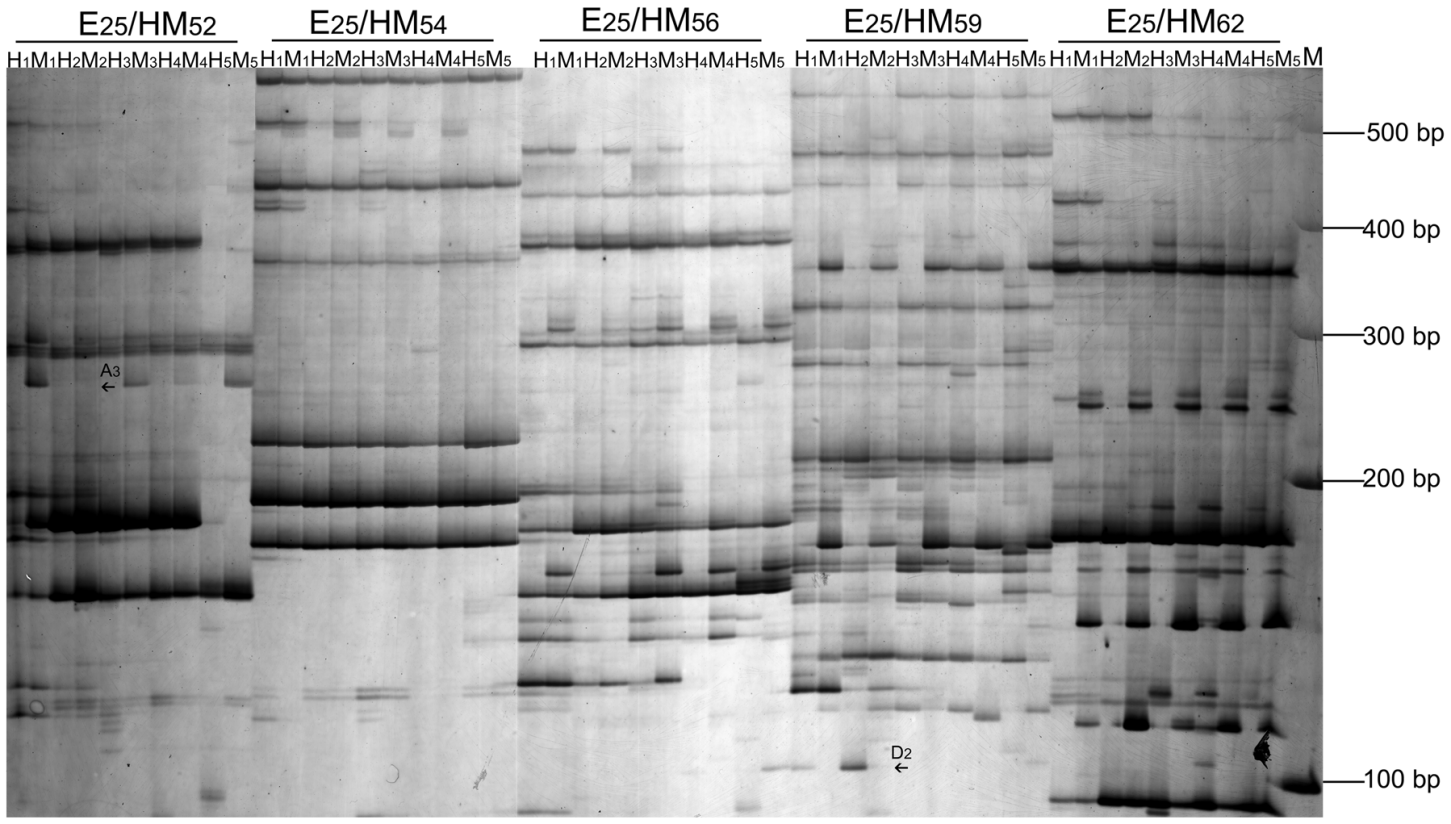
MSAP gels electrophoresis of PaWB seedlings with MMS treatment. H_1_ and M_1_: bands digested by *Eco*RI/*Hpa*II (H) and *Eco*RI/*Msp*I (M) in PS; H_2_ and M_2_: bands digested by H and M in PS-20; H_3_ and M_3_: bands digested by H and M in PS-60; H_4_ and M_4_: bands digested by H and M in PS-100; H_5_ and M_5_: bands digested by H and M in HS; E_25_/HM_52_ – E_25_/HM_x_: primer combination; M: Marker; The arrows only indicated part of the methylation patterns between PS and PS-20 (H_1_, M_1_, H_2_, M_2_).

Abundant DNA methylation patterns were detected by 96 pairs of MSAP primer combinations in the PS treated with MMS or HS (Table 3). The DNA methylation and demethylation polymorphisms increased with MMS concentration increasing (Figure 4) (Table 4), respectively. DNA methylation polymorphisms of PS-20, PS-60 and PS-100 were 16.75%, 17.37% and 17.76%, respectively, simultaneously, DNA demethylation polymorphisms were 7.14%, 7.66% and 11.11%, respectively. These results demonstrated that there exist DNA methylation and DNA demethylation events in the process of morphological changes, and more DNA methylation events than DNA demethylation events occurred. A similar trend was detected in the HS. These observations suggested that changes of DNA methylation patterns were closely related to morphological changes of Paulownia.

**Table 3:** Changes of DNA methylation patterns in PaWB seedlings with MMS treatment.

.Digestion^a^				Changes of methylation patterns		Number of differences bands^b^				Types of methylation pattern
H_1_	M_1_	H_x_	M_x_	Witches' broom	Treatment or HS	0–20	0–60	0–100	0–HS	
1	1	0	1	CCGG	CCGG	103	103	119	69	A_1_
				GGCC	GGCC					
1	1	1	0	CCGG	CCGG CCGG	89	115	117	67	A_2_
				GGCC	GGCC GGCC					
0	1	0	0	CCGG	CCGG	85	127	123	155	A_3_
				GGCC	GGCC					
1	0	0	0	CCGG CCGG	CCGG	145	117	111	149	A_4_
				GGCC GGCC	GGCC					
0	1	1	1	CCGG	CCGG	33	29	79	53	B_1_
				GGCC	GGCC					
1	0	1	1	CCGG	CCGG CCGG	25	11	49	51	B_2_
				GGCC	GGCC GGCC					
0	0	0	1	CCGG	CCGG	65	85	71	153	B_3_
				GGCC	GGCC					
0	0	1	1	CCGG	CCGG	57	81	95	157	B_4_
				GGCC	GGCC					
0	1	1	0	CCGG	CCGG CCGG	5	12	16	15	C
				GGCC	GGCC GGCC					
1	1	1	1	CCGG	CCGG	1505	1589	1563	997	D_1_
				GGCC	GGCC					
1	0	1	0	CCGG CCGG	CCGG CCGG	67	63	33	89	D_2_
				GGCC GGCC	GGCC GGCC					
0	1	0	1	CCGG	CCGG	341	327	271	381	D_3_
				GGCC	GGCC					

a : H_1_ and M_1_: bands digested by *Eco*RI/*Hpa*II (H) and *Eco*RI/*Msp*I (M) in PS; H_x_ and M_x_: bands digested by H and M in MMS treated seedlings or HS; C and CC: cytosine methylation;

b : 0–20: the number of DNA methylation patterns of PS-20 relative to PS; 0–60: the number of DNA methylation patterns of PS-60 relative to PS; 0–100: the number of DNA methylation patterns of PS-100 relative to PS; 0–HS: the number of DNA methylation patterns of HS relative to PS.

**Table 4:** DNA methylation status in PaWB seedlings with MMS treatment.

Combination	Total methylated bands^a^	Type A^b^		Type B^c^		Type C^d^		Type D^e^	
		Bands	Ratio/%	Bands	Ratio/%	Bands	Ratio/%	Bands	Ratio/%
0–20	2520	422	16.75	180	7.14	5	0.20	1913	75.91
0–60	2659	462	17.37	206	7.75	12	0.45	1979	74.43
0–100	2647	470	17.76	294	11.11	16	0.60	1867	70.53
0–HS	2336	440	18.84	414	17.72	15	0.64	1467	62.80

a : Total methylation bands  =  band of type A+ band of type B+ band of type C+ band of type D;

b : type A, DNA methylation type, type A (%)  =  (band of type A)/(total methylation bands)×100;

c : type B, DNA demethylation type, type B (%)  =  (band of type B)/(total methylation bands)×100;

d : type C, uncertain DNA methylation type, type C (%)  =  (band of type C)/(total methylation bands)×100;

e : type D, DNA methylation monomorphism, type D (%)  =  (band of type D)/(total methylation bands)×100.

### Analysis of polymorphic fragment sequences

Eighty-one clear and reproducible methylated fragments related to PaWB through the comparison of DNA methylation patterns in different seedlings were sequenced, of which 36 (44.44%) represented unannotated sequences, forty-five (55.56%) fragments were homologous with annotated sequences (Table S4), these genes encoded proteins with a variety of functions, including substance metabolism, transcription, pathogen defense and signal transduction.

### Expression analysis of polymorphic fragments

**Figure 5:**
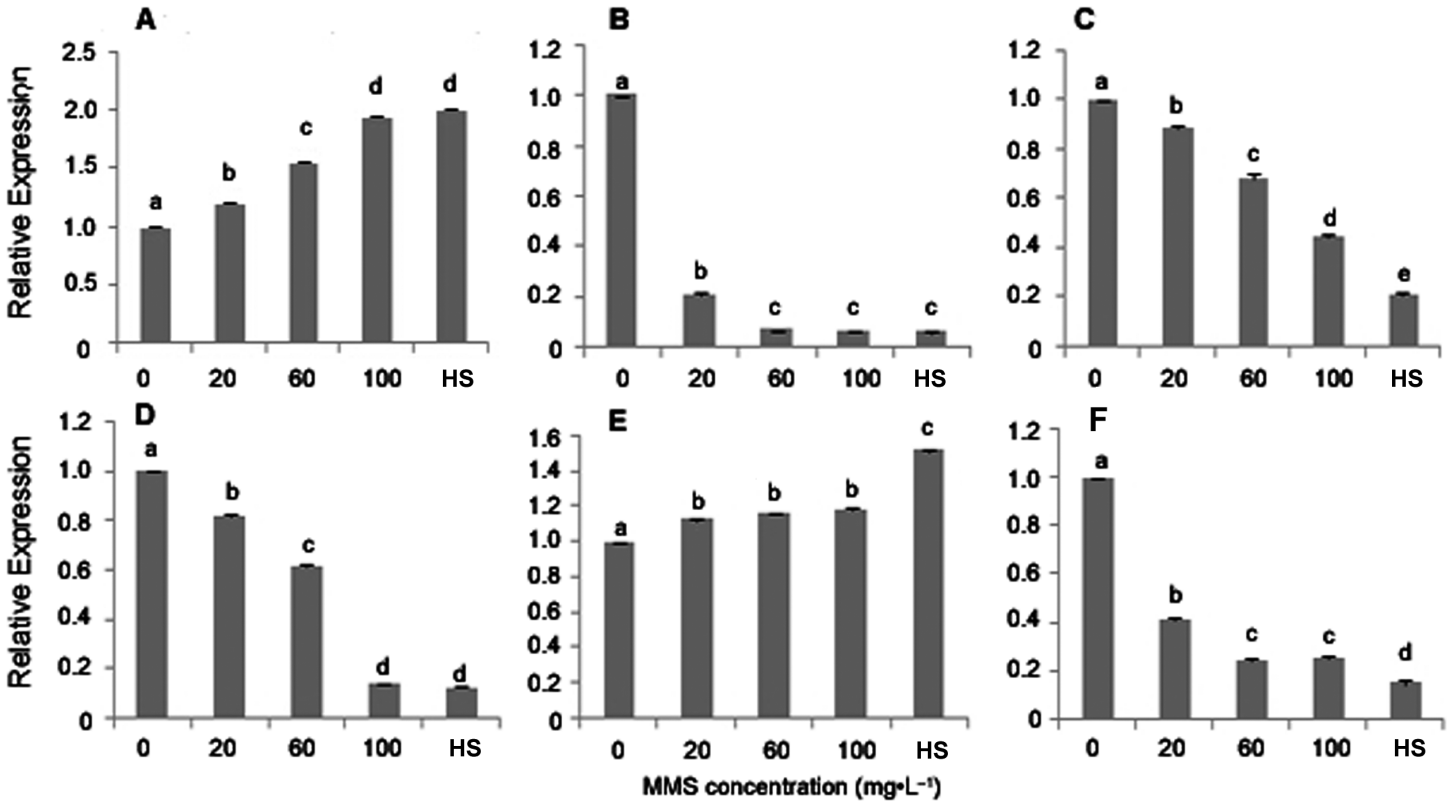
Transcriptional analysis of 6 Paulownia genes. A: relative expression of chitin-inducible gibberellin-responsive protein (Chitin-P); B: relative expression of leucyl aminopeptidase (Leucyl-A); C: relative expression of cytochrome P450 76B6 (P450); D: relative expression of ring finger protein (RFP); E: relative expression of uncharacterized protein LOC100796964 (UP); F: relative expression of beta-hydroxyacyl-ACP dehydrase 1(Beta-ACP). 0–100: MMS concentrations (mg·L^−1^); HS: healthy seedlings. The different letters within a gene repression level indicate significant difference, while the same letters within a gene repression level indicate no significant differences (*p*<0.05).

The expressions of six methylated genes were analyzed using qRT-PCR. The results showed that two genes encoding proteins (chitin-inducible gibberellin-responsive protein and uncharacterized protein LOC100796964) (Figure 5A and E) were up-regulated and four genes encoding proteins (leucyl aminopeptidase, cytochrome P450 76B6, ring finger protein and beta-hydroxyacyl-ACP dehydrase 1) (Figure 5B, C, D and F) were down-regulated with MMS concentration increasing (*p*<0.05), indicating that the expressions of all six genes were consistent with the changes of DNA methylation patterns.

## Discussion

The changes of DNA polymorphism and DNA methylation in the PS, the ones treated with MMS and HS, based on AFLP and MSAP approaches in this paper showed that phytoplasma infection did not change the DNA sequence of seedlings at AFLP level, but changed the DNA methylation levels and patterns, and the observation from AFLP was consistent with the previous results [7], [18], [19], but differed from the TMV-infected tobacco [28] in which the host exhibited an increase in the homologous recombination frequency (HRF), implying that the morphological changes of the seedlings treated with MMS may be related to the epigenetic modifications. DNA methylation is one of the main epigenetic modifications, plays a vital role in plant growth and development, changes of DNA methylation levels has closely related to plant phenotypic changes, the DNA methylation pattern, such as DNA hypermethylation or hypomethylation can result in their morphological abnormalities [29], [30]. The MSAP analysis showed that DNA methylation level of PS was lower than those treated with MMS and HS. This finding is in agreement with our previous HPLC result [16], demonstrating that the decrease of host DNA methylation levels was related to PaWB. This coincides with the results in *Arabidopsis* [31], [32], 5-azacytidine treated brassica-oleracea [33] and ‘*Candidatus* Phytoplasma asteris’-infected periwinkles [34]. Moreover, variations of DNA methylation patterns were also discovered when the PS acquired a healthy morphology. The DNA methylation or demethylation polymorphisms increased with MMS concentration increasing. Although DNA demethylation polymorphisms also occurred at a high frequency, the DNA methylation polymorphisms were generally the most frequent, these results were consistent with the previous observations [19], suggesting that the variations of DNA methylation patterns are a dynamic process. These results indicated that the occurrence of PaWB is a complex process, morphological changes of Paulownia after phytoplasma infection has relation with DNA methylation.

Plant responds to pathogen attack by modifying gene expression. DNA methylation is one of the mechanisms in regulating gene expressions [30], similar results were also reported in Tomato Yellow Leaf Curl Sardinia Virus-Tomato [14] and *Mycosphaerella fijiensis* toxins-Musa [35] systems. In the present study, we identified several DNA methylation genes related to PaWB, and qRT-PCR showed that their expressions significantly changed in the process of the PS morphological changes. Among these genes, their functions not only involved in phytoplasma virulence, but also implicated in symptom formation and disease defense of PaWB. The genes encoded leucyl aminopeptidase implicated in phytoplasma vitamine metabolic pathways [36], so the higher expression of leucyl aminopeptidase was related to the phytoplasma virulence; The gene encoded beta-hydroxyacyl-ACP dehydrase 1 involved in fatty acid biosynthesis pathway [10], [37], which is necessary for phytoplasma [38], so the higher gene expression of these genes in the PS may be related to phytoplasma overgrowth, this means that over-expression of these genes further disturbed the normal growth of Paulownia, and resulted in the changes of set of genes expressions. For example, the gene encoding chitin-inducible gibberellin-responsive protein (CIGR) was down-regulation in the PS, it was reported that CIGR belongs to the GRAS family, mainly contributes to the stem elongation of plant [39], so the lower expression of this gene in the PS might correlate with the dwarf of the Paulownia; The gene encoding cytochrome P450 76B6 was up-regulation in the PS, which consistent with the global transcriptome result [9], [10]. Previous research have evidenced that cytochrome P450 76B6 played an important role in biosynthesis of flavonoids [40], which had closely related to decrease of the leaf cell death after phytoplasma infection [41], numerous studies have revealed that flavonoids were induced in response to pathogen infection [42]–[45], so the higher expression of this gene in the phytoplasna infection seedlings might implicate in plant defense; Another protein involved in plant defense was ring finger protein [46], the trend of this gene expression in the PS and the ones treated with MMS was similar with that reported by Mou et al. [9]. Overall, morphological changes of Paulownia seedlings after phytoplasma infection resulted in various changes of the DNA methylation patterns related to PaWB, which further induced the changes of corresponding gene expressions in the PS and the ones treated with MMS, interesting, several genes were detected by both DNA methylation and transcriptome analysis. Besides these genes, we also found 36 unannotated genes whose functions were not clear and worthy of further investigation in future studies.

The occurrence of PaWB involved in many factors. Even though several genes involved in metabolic pathways of Paulownia had been identified, but a few of these genes might only be associated with the growth and development of *P. fortunei* itself. The similar result was got in *Paulownia tomentosa × Paulownia fortunei* [19]. In order to identify some genes more closely related to the occurrence of PaWB, the genes associated with the growth and development had to be discarded. Through comparing the sequences and the sizes of the methylation genes generated in *P. fortunei* and *P. tomentosa × P. fortunei*, we found that three genes appeared simultaneously in two species of Paulownia seedlings with PaWB, including chase 2 sensor protein, cation proton exchanger, and transcription factor HB29. The roles of these three genes in the process of occurrence of PaWB retain unknown, we will put emphasis on them in our next research.

In conclusion, phytoplasma infection resulted in the *P. fortunei* morphological changes of the seedlings, but these changes could be recovered by more than 60 mg·L^−1^MMS treatment. DNA polymorphisms analysis showed that Paulownia DNA sequence was not changed in the process of morphological changes at AFLP level, conversely, these variations regulated by changes of DNA methylation levels and patterns, providing further clues to clarify the molecular mechanism of PaWB.

## Supporting Information

Figure S1 **AFLP gels electrophoresis of PaWB seedlings with MMS treatment.** a: bands amplification obtained from PS; b: bands amplification obtained from PS-20; c: bands amplification obtained from PS-60; d: bands amplification obtained from PS-100; e: bands amplification obtained from HS; M: DNA Marker; P_1_/M_21_ – P_1_/M_x_: primer combinations.
(TIF)

Table S1 **AFLP adapters and primers used in this study.** P_1_/M_1_–P_64_/M_64_ are the selective-amplification primer combinations.
(DOCX)

Table S2 **MSAP adapters and primers used in this study.** ^*^Selective-amplification primer combinations comprised each *Eco*RI primer combined with each *Hpa*II/*Msp*I primer.
(DOCX)

Table S3 **Primers used for qRT - PCR analysis.**
(DOCX)

Table S4 **List of MSAP fragment with different methylation profiles in PaWB seedlings with MMS treatment.** ^a^: BB1–BB52: MSAP polymorphic fragments during PS, MMS treated PS and HS; ^b^: the sequence information obtained from the GenBank database.
(DOCX)
