# Supplementary figures and images for: Morphological Changes of Paulownia Seedlings Infected Phytoplasmas Reveal the Genes Associated with Witches' Broom through AFLP and MSAP
Source: PLoS One. 2014 Nov 26;9(11):e112533. doi: 10.1371/journal.pone.0112533 (PMC4245194; doi:10.1371/journal.pone.0112533)

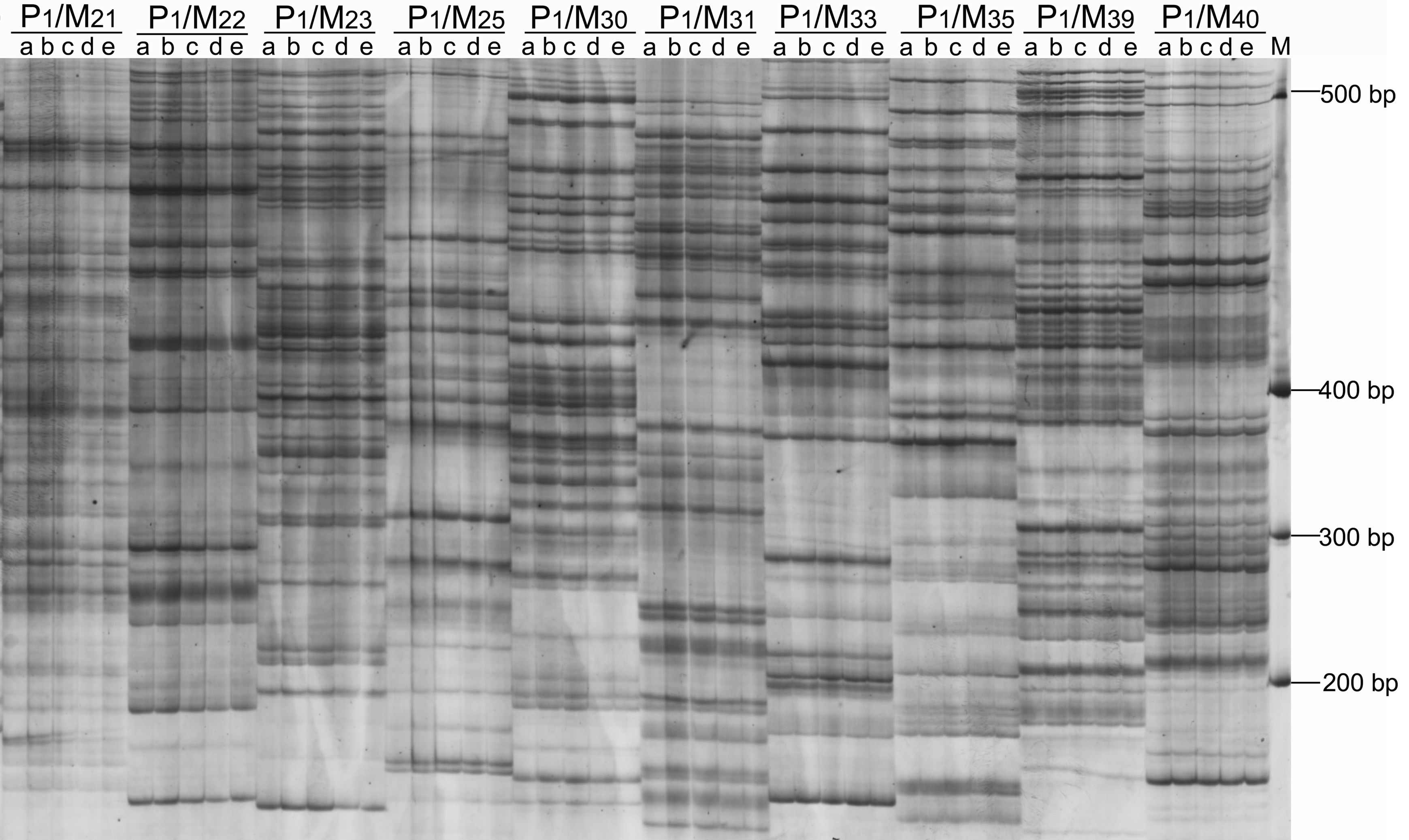

Supplement: Figure S1 — AFLP gels electrophoresis of PaWB seedlings with MMS treatment. a: bands amplification obtained from PS; b: bands amplification obtained from PS-20; c: bands amplification obtained from PS-60; d: bands amplification obtained from PS-100; e: bands amplification obtained from HS; M: DNA Marker; P1/M21 – P1/Mx: primer combinations. (TIF) [file pone.0112533.s001.tif]
