# Supplementary material for: Morphological Changes of Paulownia Seedlings Infected Phytoplasmas Reveal the Genes Associated with Witches' Broom through AFLP and MSAP
Source: PLoS One. 2014 Nov 26;9(11):e112533. doi: 10.1371/journal.pone.0112533 (PMC4245194; doi:10.1371/journal.pone.0112533)
Supplement: Table S1 — AFLP adapters and primers used in this study. P1/M1–P64/M64 are the selective-amplification primer combinations. (DOCX) [file pone.0112533.s002.docx]

**Table S1 AFLP adapters and primers used in this study**

| Name | Sequence (5' - 3') | | | | |
| --- | --- | --- | --- | --- | --- |
| Adaptors | *Pst* I-F:CTCGTAGACTGCGTACATGCA; *Pst* I-R:TGTACGCAGTCTAC; *Mse* I-F:GACGATGAGTCCTGAG; *Mse* I-R:TACTCAGGACTCAT | | | | |
| Pre-amplification primer | *Pst* I (P):GACTGCGTACATGCAG; *Mse* I (M):GATGAGTCCTGAGTAA | | | | |
| Selective-amplification primer combinations | P+AAA/M+AAA(P_1_/M_1_) | P+AAC/M+AAG(P_1_/M_2_) | P+AAA/M+AAC(P_1_/M_3_) | P+AAA/M+AAT(P_1_/M_4_) | P+AAA/M+ACC(P_1_/M_11_) |
|  | P+AAA/M+ACT(P_1_/M1_2_) | P+AAC/M+ATA(P_1_/M_13_) | P+AAA/M+ATG(P_1_/M_14_) | P+AAA/M+ATC(P_1_/M_15_) | P+AAA/M+ATT(P_1_/M_16_) |
|  | P+AAA/M+GAG(P_1_/M_18_) | P+AAT/M+GAC(P_1_/M_19_) | P+AAA/M+GGA(P_1_/M_21_) | P+AAA/M+GGG(P_1_/M_22_) | P+AAA/M+GGC(P_1_/M_23_) |
|  | P+AAA/M+GCA(P_1_/M_25_) | P+AGA/M+GTG(P_1_/M_30_) | P+AAA/M+GTC(P_1_/M_31_) | P+AAA/M+CAA(P_1_/M_33_) | P+AAA/M+CAC(P_1_/M_35_) |
|  | P+AAA/M+CGC(P_1_/M_39_) | P+AGG/M+CGT(P_1_/M_40_) | P+AAA/M+CTG(P_1_/M_46_) | P+AAA/M+CTC(P_1_/M_47_) | P+AAA/M+CTT(P_1_/M_48_) |
|  | P+AAA/M+TGA(P_1_/M_53_) | P+AGG/M+AGG(P_1_/M_54_) | P+AAA/M+TGT(P_1_/M_56_) | P+AAG/M+ACA(P_2_/M_9_) | P+AAG/M+ACC(P_2_/M_11_) |
|  | P+AAG/M+ACT(P_2_/M_12_) | P+AAG/M+ATC(P_2_/M_15_) | P+AAA/M+GAA(P_2_/M_17_) | P+AAG/M+GGA(P_2_/M_21_) | P+AAG/M+GGG(P_2_/M_22_) |
|  | P+AAG/M+GGC(P_2_/M_23_) | P+AAG/M+GCA(P_2_/M_25_) | P+AAG/M+GCT(P_2_/M_28_) | P+AAG/M+GTT(P_2_/M_32_) | P+AAG/M+CAC(P_2_/M_35_) |
|  | P+AAG/M+CAT(P_2_/M_36_) | P+AAG/M+CTG(P_2_/M_46_) | P+AAG/M+CTC(P_2_/M_47_) | P+AAG/M+TAA(P_2_/M_49_) | P+AAG/M+TTT(P_2_/M_64_) |
|  | P+AAC/M+AGA(P_3_/M_5_) | P+AAC/M+AGC(P_3_/M_7_) | P+AAC/M+ACT(P_3_/M_12_) | P+AAC/M+ATA(P_3_/M_13_) | P+AAC/M+ATG(P_3_/M_14_) |
|  | P+AAC/M+GAA(P_3_/M_17_) | P+AAC/M+GAT(P_3_/M_20_) | P+AAC/M+GCG(P_3_/M_26_) | P+AAC/M+TAG(P_3_/M_50_) | P+AAC/M+TGG(P_3_/M_54_) |
|  | P+AAC/M+TCA(P_3_/M_57_) | P+AAC/M+TCC( P_3_/M_59_) | P+AAC/M+TCT(P_3_/M_60_) | P+AAC/M+TTC(P_3_/M_63_) | P+AAT/M+GCA(P_4_/M_25_) |
|  | P+AAT/M+GTG(P_4_/M_30_) | P+AAT/M+CTC(P_4_/M_47_) | P+AAT/M+CTT(P_4_/M_48_) | P+AAT/M+TAC(P_4_/M_51_) | P+AAA/M+AGA(P_5_/M_5_) |
|  | P+AGA/M+ACA(P_5_/M_9_) | P+AGA/M+ATA(P_5_/M_13_) | P+AGA/M+ATC(P_5_/M_15_) | P+AGA/M+ATT(P_5_/M_16_) | P+AGA/M+GTG(P_5_/M_30_) |
|  | P+AGA/M+CAG(P_5_/M_34_) | P+AGA/M+CAT(P_5_/M_36_) | P+AGA/M+CCA(P_5_/M_41_) | P+AGA/M+CTA(P_5_/M_45_) | P+AGA/M+CTG(P_5_/M_46_) |
|  | P+AGA/M+CTT(P_5_/M_48_) | P+AGA/M+TAA(P_5_/M_49_) | P+AGA/M+TAT(P_5_/M_52_) | P+AGA/M+TGC(P_5_/M_55_) | P+AGA/M+TCC(P_5_/M_59_) |
|  | P+AGA/M+TCT(P_5_/M_60_) | P+AGA/M+TTA(P_5_/M_61_) | P+AGG/M+AAA(P_6_/M_1_) | P+AGG/M+ACA(P_6_/M_9_) | P+AGG/M+ATG(P_6_/M_14_) |
|  | P+AGG/M+ATT(P_6_/M_16_) | P+AGG/M+GTT(P_6_/M_32_) | P+AGG/M+TAC(P_6_/M_51_) | P+AGG/M+TCA(P_6_/M_57_) | P+AGG/M+TCT(P_6_/M_60_) |
|  | P+AGG/M+GGA(P_8_/M_21_) | P+AGG/M+CAA(P_8_/M_33_) | P+AGG/M+TAT(P_8_/M_52_) | P+AGG/M+TCA(P_8_/M_57_) | P+TTC/M+TTC(P_63_/M_63_) |
|  | P+TTT/M+TTT(P_64_/M_64_) |  |  |  |  |

P_1_/M_1_-----P_64_/M_64_ are numbers of selective-amplification primer combinations.
