# Supplementary material for: Morphological Changes of Paulownia Seedlings Infected Phytoplasmas Reveal the Genes Associated with Witches' Broom through AFLP and MSAP
Source: PLoS One. 2014 Nov 26;9(11):e112533. doi: 10.1371/journal.pone.0112533 (PMC4245194; doi:10.1371/journal.pone.0112533)
Supplement: Table S2 — MSAP adapters and primers used in this study. *Selective-amplification primer combinations comprised each EcoRI primer combined with each HpaII/MspI primer. (DOCX) [file pone.0112533.s003.docx]

**Table S2 MSAP adapters and primers used in this study**

| Name | Sequence (5'-3') | |
| --- | --- | --- |
|  | *Eco*RI (E) | *Hpa*II/*Msp*I(HM) |
| Adaptor | Adaptor –F: CTCGTAGACTGCGTACC | Adaptor –F: GATCATGAGTCCTGCT |
|  | Adaptor –R: AATTGGTACGCAGTCTAC | Adaptor –R: CGAGCAGGACTCATGA |
| Pre-amplification primer | E: GACTGCGTACCAATTCA | HM: ATCATGAGTCCTGCTCGGT |
| Selective-amplification primer^*^ | E+AAA(E1) | HM+AAC(HM4) |
|  | E+AGG(E11) | HM+ATT(HM6) |
|  | E+TAC(E20) | HM+ACA(HM13) |
|  | E+TTT(E22) | HM+TGG(HM27) |
|  | E+TGA(E25) | HM+GTC(HM40) |
|  | E+TGT(E26) | HM+GGA(HM41) |
|  | E+GAC(E36) | HM+GCC(HM48) |
|  | E+CGC(E60) | HM+CAC(HM52 |
|  |  | HM+CTT(HM54) |
|  |  | HM+CTC(HM56) |
|  |  | HM+CGG(HM59) |
|  |  | HM+CCT(HM62) |

^*^Selective-amplification primer combinations are each of the *Eco*RI primer combined with each of the *Hpa*II/*Msp*I primer.
