# Supplementary material for: Morphological Changes of Paulownia Seedlings Infected Phytoplasmas Reveal the Genes Associated with Witches' Broom through AFLP and MSAP
Source: PLoS One. 2014 Nov 26;9(11):e112533. doi: 10.1371/journal.pone.0112533 (PMC4245194; doi:10.1371/journal.pone.0112533)
Supplement: Table S3 — Primers used for qRT - PCR analysis. (DOCX) [file pone.0112533.s004.docx]

**Table S3 Primers used for qRT-PCR analysis**

| Gene name | Forward primer sequence (5‘ - 3’) | Reverse primer sequence (5‘ - 3’) |
| --- | --- | --- |
| 18S | ACATAGTAAGGATTGACAGA | TAACGGAATTAACCAGACA |
| Chitin-P | CTCTTTCCTCGCTATTAACCCTTC | GTGCTGCCGTTTCTATTAGTGG |
| Leucyl-A | TGGATCAGCAAGGTAAGTG | GAATATCGCCAGGCAGTG |
| P450 | GACGCCAACCAGCACCTGAG | GCCGCCTGTCTGACTATTCTTCC |
| RFP | TTGACCAGGCTTTGATTGATG | CGCAGACAAACAGCACAA |
| UP | CGGTATTTCCCTCGAATTTGG | ACGCCGTTTGCCTAAGTG |
| Beta-ACP | AGACACCTTAGTAATGAGAA | CACTTCACCACCTACATA |
