# Supplementary material for: Morphological Changes of Paulownia Seedlings Infected Phytoplasmas Reveal the Genes Associated with Witches' Broom through AFLP and MSAP
Source: PLoS One. 2014 Nov 26;9(11):e112533. doi: 10.1371/journal.pone.0112533 (PMC4245194; doi:10.1371/journal.pone.0112533)
Supplement: Table S4 — List of MSAP fragment with different methylation profiles in PaWB seedlings with MMS treatment. a: BB1–BB52: MSAP polymorphic fragments during PS, MMS treated PS and HS; b: the sequence information obtained from the GenBank database. (DOCX) [file pone.0112533.s005.docx]

**Table S4 List of MSAP fragments with different methylation profile in PaWB seedlings with MMS treatment**

| Number^a^ | Sequence homology^b^ | Accession No. | Positives/(%) | Expect |
| --- | --- | --- | --- | --- |
| BB1 | Chitin-inducible gibberellin-responsive protein, putative | [XP_002522814.1](http://www.ncbi.nlm.nih.gov/protein/255563625?report=genbank&log$=protalign&blast_rank=1&RID=MDAKFH1T016) | 92.68 | 2.00E-13 |
| BB2 | Chitin-inducible gibberellin-responsive protein, putative | XP_002522814.1 | 95 | 3.00E-13 |
| BB3 | Chitin-inducible gibberellin-responsive protein, putative | XP_002522814.1 | 95 | 3.00E-13 |
| BB4 | Chitin-inducible gibberellin-responsive protein, putative | XP_002522814.1 | 92.68 | 2.00E-13 |
| BB5 | Chitin-inducible gibberellin-responsive protein, putative | XP_002522814.1 | 92.68 | 2.00E-13 |
| BB6 | Chitin-inducible gibberellin-responsive protein, putative | XP_002522814.1 | 92.68 | 2.00E-13 |
| BB7 | Chase2 sensor protein | YP_004040123.1 | 100 | 8.00E-09 |
| BB8 | GYF domain-containing protein | NP_199109.1 | 90 | 1.00E-07 |
| BB9 | Leucyl aminopeptidase | YP_003050015.1 | 96.88 | 1.00E-10 |
| BB10 | Cytochrome P450 76B6 | Q8VWZ7.1 | 95.35 | 2E-18 |
| BB11 | Chitin-inducible gibberellin-responsive protein, putative | XP_002522814.1 | 97.5 | 2.00E-13 |
| BB12 | Chitin-inducible gibberellin-responsive protein, putative | XP_002522814.1 | 97.5 | 7.00E-13 |
| BB13 | Chitin-inducible gibberellin-responsive protein, putative | XP_002522814.1 | 97.5 | 6.00E-14 |
| BB14 | Chitin-inducible gibberellin-responsive protein, putative | XP_002522814.1 | 92.68 | 2.00E-13 |
| BB15 | Chitin-inducible gibberellin-responsive protein, putative | XP_002522814.1 | 97.5 | 2.00E-13 |
| BB16 | Chitin-inducible gibberellin-responsive protein, putative | XP_002522814.1 | 97.5 | 2.00E-13 |
| BB17 | Electron transporter, putative | XP_002522814.1 | 82.35 | 1.00E-13 |
| BB18 | Predicted protein | XP_002310215.1 | 100 | 0.001 |
| BB19 | PREDICTED: zinc finger protein ZAT5-like isoform 1 | XP_004250033.1 | 100 | 1.00E-08 |
| BB20 | PREDICTED: zinc finger protein ZAT5-like isoform 1 | XP_004250033.1 | 100 | 1.00E-08 |
| BB21 | PREDICTED: zinc finger protein ZAT5-like isoform 1 | XP_004250033.1 | 100 | 1.00E-08 |
| BB22 | Cytochrome b/b6 protein | YP_003047764.1 | 100 | 6.00E-10 |
| BB23 | Cytochrome b/b6 protein | YP_003050059.1 | 100 | 1.00E-10 |
| BB24 | Cytochrome b/b6 protein | YP_003050059.1 | 100 | 1.00E-09 |
| BB25 | Cytochrome b/b6 protein | YP_003050059.1 | 100 | 1.00E-09 |
| BB26 | Cytochrome b/b6 protein | YP_003050059.1 | 100 | 1.00E-09 |
| BB27 | PREDICTED: homeobox-leucine zipper protein ATHB-7-like | XP_004245456.1 | 88 | 8.00E-04 |
| BB28 | Hypothetical protein Msip34_0503 | YP_003050278.1 | 96.39 | 2.00E-50 |
| BB29 | Hypothetical protein Msip34_0503 | YP_003050278.1 | 96.39 | 2.00E-50 |
| BB30 | Ring finger protein, putative | XP_002511433.1 | 97.06 | 2E-15 |
| BB31 | Beta-hydroxyacyl-ACP dehydrase 1 | ACJ07147.1 | 94.74 | 2E-16 |
| BB32 | Beta-hydroxyacyl-ACP dehydrase 1 | ACJ07147.1 | 94.74 | 2E-16 |
| BB33 | Beta-hydroxyacyl-ACP dehydrase 1 | ACJ07147.1 | 94.74 | 2.00E-16 |
| BB34 | Ring finger protein, putative | XP_002318032.1 | 91.18 | 3.00E-13 |
| BB35 | Ring finger protein, putative | XP_002511433.1 | 97.06 | 2.00E-15 |
| BB36 | PREDICTED: RING-H2 finger protein ATL47-like | XP_003517782.1 | 97.06 | 3.00E-15 |
| BB37 | Ring finger protein, putative | XP_002511433.1 | 97.06 | 2.00E-15 |
| BB38 | Predicted protein | XP_002319305.1 | 91.3 | 3.00E-07 |
| BB39 | Guanylate kinase | YP_003049827.1 | 100 | 4.00E-07 |
| BB40 | Cation proton exchanger | XP_002297994.1 | 70.73 | 1.00E-05 |
| BB41 | Beta-hydroxyacyl-ACP dehydrase 1 | ACJ07147.1 | 94.74 | 2.00E-16 |
| BB42 | Beta-hydroxyacyl-ACP dehydrase 1 | ACJ07147.1 | 94.74 | 2.00E-16 |
| BB43 | PREDICTED: uncharacterized protein LOC100796964 | XP_003544978.1 | 95.65 | 2.00E-07 |
| BB44 | Ferrichrome-binding protein | ZP_03104379.1 | 100 | 4.00E-07 |
| BB45 | Predicted protein | XP_002319305.1 | 91.3 | 3.00E-07 |
| BB46 | Beta-hydroxyacyl-ACP dehydrase 1 | ACJ07147.1 | 94.74 | 2.00E-16 |
| BB47 | Dihydrodipicolinate synthetase | YP_004039809.1 | 97.3 | 3.00E-18 |
| BB48 | Dihydrodipicolinate synthetase | YP_004039809.1 | 97.22 | 5.00E-17 |
| BB49 | PREDICTED: uncharacterized protein LOC101260367 | XP_004245445.1 | 89.29 | 8.00E-08 |
| BB50 | Transcription factor HB29 | Q9SEZ1.1 | 88.89 | 4.00E-08 |
| BB51 | Eukaryotic translation initiation factor 2c, putative | XP_002527383.1 | 82.76 | 7.00E-05 |
| BB52 | Eukaryotic translation initiation factor 2c, putative | XP_004243504.1 | 96 | 4.00E-06 |

^a^: BB1–BB52: MSAP polymorphic fragments during PS, MMS treated PS and HS; ^b^: The sequence information obtained from the GenBank database.
